# Influence of anthranilic diamide insecticides on emerald ash borer (Coleoptera: Buprestidae) larvae and establishment of introduced parasitoids in central Kentucky

**DOI:** 10.1093/jisesa/ieag103

**Published:** 2026-09-16

**Authors:** Caleb J Wilson, Zoë K York

**Affiliations:** Department of Entomology, University of Kentucky, Lexington, KY, USA; Department of Entomology, University of Kentucky, Lexington, KY, USA

**Keywords:** emerald ash borer, anthranilic diamide, biological control, parasitoid wasp, ash

## Abstract

Emerald ash borer (EAB) (*Agrilus planipennis* Fairmaire) (Coleoptera: Buprestidae) is an invasive pest of ash trees (*Fraxinus* spp.) in the United States. EAB is best managed via insecticides and biological control by wasps and woodpeckers. Neonicotinoids are often used for EAB due to cost and ease of use but are becoming restricted worldwide and are toxic to beneficial insects. Anthranilic diamides, a newer insecticide class, can manage beetle larvae with limited off-target effects. If diamides control EAB, they could replace neonicotinoids. We tested if drenches of chlorantraniliprole and cyantraniliprole at 3 rates controlled EAB larvae without affecting parasitoids in 41 recruit-sized white ash, *Fraxinus americana* L. (mean ± SE = 11.0 ± 0.7 cm diameter at breast height (DBH)) in central Kentucky 6 months after application. Only the high chlorantraniliprole rate (0.157 g a.i./L per cm DBH) significantly reduced EAB densities (1.3 ± 0.5 larvae per m^2^) relative to controls (9.5 ± 4.2 larvae per m^2^). Three treatments reduced parasitism by *Phasgonophora sulcata* Westwood relative to controls while no treatment affected nonnative parasitism or woodpecker predation. Thirty-six percent of larvae were parasitized even though the nearest parasitoid releases were over 20 km away and occurred 7 years prior. *Spathius galinae* Belokobylskij & Strazanac parasitism, never released in Kentucky at the time of this project, averaged 7.8 ± 2.9 % and is the furthest south this species has established in the United States. Chlorantraniliprole may control EAB in small diameter ash and reduce parasitism by *P. sulcata* but not introduced parasitoids.

## Introduction

Emerald ash borer (EAB) (*Agrilus planipennis* Fairmaire) (Coleoptera: Buprestidae) is an invasive phloem-feeding beetle that has killed millions of ash trees across eastern North America (Liebhold et al. 2013) which continues to spread westward (Zhu et al. 2025). EAB can also infest white fringe trees (*Chionanthus virginicus* L.) and olive trees (*Olea europaea* L.), although both species are more resistant than most ash species (Peterson and Cipollini 2017, Cipollini 2025). Management interventions for EAB include chemical control, natural biological control by woodpeckers, and classical biological control with introduced parasitoid wasps (McCullough 2020).

The most effective chemical control for EAB is trunk injection of emamectin benzoate every 2 to 3 years (McCullough et al. 2019, Sadof et al. 2023). Other effective insecticides include azadirachtin, and the neonicotinoids imidacloprid, and dinotefuran, but they require annual reapplication to remain effective (McKenzie et al. 2010, Smitley et al. 2010, 2015, McCullough et al. 2011, 2019). Despite their shorter residual time, neonicotinoids are widely used to manage EAB because they are easier to apply and cheaper than emamectin benzoate. However, neonicotinoids are becoming increasingly restricted worldwide, necessitating other options to manage EAB in locations where restrictions exist (Sharma and Sanyal 2024, Dentzman et al. 2025). Neonicotinoids are also toxic to beneficial insects (Main et al. 2018) and may kill nontarget taxa within treated ash, as has been speculated for eastern hemlock trees (*Tsuga canadensis*) treated for hemlock wooly adelgid (*Adelges tsugae* Annand) (Frank and Tooker 2020).

In the United States, four introduced parasitoid wasp species are regularly released for EAB biological control, and where established, reduce larval densities in infested trees by roughly one-third to one half (Duan et al. 2023a). Few studies have measured establishment of EAB parasitoids in the southern United States, even though releases have been occurring in this region for ca. 10 years (Davidson and Rieske 2016, MapBioControl.org 2026). When testing new insecticide classes on EAB, potential impacts to introduced parasitoids should also be assessed to ensure compatibility.

Anthranilic diamide insecticides are widely used to manage pests of lawns and woody plants. They generally have fewer off-target effects in comparison to neonicotinoids (Larson et al. 2012, 2013, Redmond and Potter 2017). Diamides activate insect ryanodine receptors and cause an uncontrolled release of Ca^2+^ ions from the sarcoplasmic reticulum within muscle cells which results in paralysis and death of affected insects (Cordova et al. 2006, Sahoo et al. 2025). Diamides have systematic activity within plants and are often applied as foliar sprays or soil drenches, typically to manage root or foliage feeding pests (e.g. Tiwari and Stelinski 2013, Redmond and Potter 2017). If effective on EAB, diamide soil drenches may be an easy way for foresters, arborists, and homeowners to protect trees without specialized equipment.

Redmond and Potter (2017) found that soil drenches of chlorantraniliprole failed to control boxwood psyllid [*Cacopsylla busi* (L.)], and boxwood leafminer [*Monarthropalpusi flavus* (Schank)] on boxwood (*Buxus sempirvirens* L.), but resulted in 70% reduction of rose slug sawfly [*Endelomyia aethiops* (Fabricius)] herbivory and significantly reduced Japanese beetle (*Popillia japonica* Newman) herbivory on roses (*Rosa* “Knock out”) 6 to 7 months after application. Chlorantraniliprole reduced herbivory by black vine weevil (*Otiorhynchus sulcatus* Fabricius) on potted *Taxus* shrubs for 12 days without influencing adult mortality and reduced larval numbers by 92% in potted *Sedum* plants (Reding and Persad 2009, Reding and Ranger 2011). McCarty et al. (2023) found that chlorantraniliprole protected loblolly pine (*Pinus taeda* L.) seedlings from Nantucket pine tip moth (*Rhyacionia frustrana* Scudder *in* Comstock) for 2 years. The authors also noted that trees treated with chlorantraniliprole were healthier after 2 years than trees treated with imidacloprid, dinotefuran, or fipronil (McCarty et al. 2023). Finally, Tiwari and Stelinski (2013) found that soil drench applications of cyantraniliprole on 5-year-old “Navel” citrus trees significantly reduced Asian citrus psyllid (*Diaphorina citri* Kuwayama) and citrus leaf miner (*Phyllocnistis citrella* Stainton) counts relative to control and thiamethoxam treatments.

Most studies have tested diamides on pests in small trees or shrubs, and no study has evaluated impacts on any wood boring pest. Chlorantraniliprole and cyantraniliprole have reduced solubility, and thus, systematic activity, when compared with neonicotinoids (Bonmatin et al. 2015, Lahm et al. 2019) which has been postulated as a reason for limited efficacy on pests such as black vine weevil in comparison to neonicotinoids (Reding and Ranger 2011). Since uptake and translocation of systemic insecticides within plants can vary across species (Cloyd et al. 2011), it is unclear if diamides would translocate effectively within ash and reach developing EAB larvae, or if translocation would be affected by tree size.

USDA APHIS has been releasing Asian EAB parasitoids in North America since 2007 (Duan et al. 2022). While these introduced species have established in many areas invaded by EAB, there has been considerable spatiotemporal variation in establishment success and parasitism rates (Duan et al. 2012, Quinn et al. 2022a, 2022b, Wilson et al. 2024, MapBioControl.org 2026). For example, Wilson et al. (2024) recorded EAB parasitism rates by *Spathius galinae* of 11% in heavily infested pole-sized trees (DBH: 11 to 15 cm), whereas Duan et al. (2023b) found parasitism rates of 43% to 60% by this species in the same region of south-central Michigan in trees with DBH 9 to 13 cm. Nonetheless, where long-term releases of introduced EAB parasitoids have occurred, parasitism rates generally increased over time (Duan et al. 2022, 2023a, 2023b). There are also many native parasitoid species that attack EAB larvae (Davidson and Rieske 2015). Although most native taxa usually do not parasitize over 5% of larvae within infested trees (Bauer et al. 2015, Davidson and Rieske 2015), *Atanycolus cappaerti* Förster (Braconidae) and *Phasgonophora sulcata* Westwood (Chalcididae) have been recorded parasitizing up to 80% and 34% of EAB larvae, respectively (Cappaert and McCullough 2009, Roscoe et al. 2016). Most studies on EAB parasitoids have occurred in the Midwest and northeast, whereas few have measured parasitoid establishment and impact on EAB in the southern United States, where climactic differences can alter EAB and parasitoid phenology (Ragozzino et al. 2020, Bohannon et al. 2022).

Since EAB parasitoid releases may occur in forest stands or landscapes where ash are treated with insecticides, it is important to characterize potential impacts of treatments on parasitoid establishment and efficacy. The goal of this study was (i) to determine if anthranilic diamides can control EAB, (ii) if anthranilic diamides are compatible with EAB biological control agents, and (iii) measure introduced parasitoid wasp establishment in postinvasion ash stands in central Kentucky. Because diamides can control beetle larvae (e.g. Koppenhöfer et al. 2019) and since chlorantraniliprole has previously been shown to not harm bees or ants (Larson et al. 2012, 2013), we hypothesized that diamide treatments would control EAB while not impairing biological control by parasitoids. We tested the effect of 2 anthranilic diamides (chlorantraniliprole and cyantraniliprole) applied as soil drenches at various rates on EAB larvae and parasitism, and measured introduced parasitoid establishment at a site where the nearest parasitoid releases had been >20 km away and occurred 7 years prior.

## Methods

On 19 July 2024, we located 42 white ash at Taylor Fork Ecological Area (TFEA) in Richmond, Kentucky (37.714683, −84.295768) to test the effect of 3 different rates of 2 different anthranilic diamides, chlorantraniliprole (Durentis; FMC Corporation, Philadelphia, Pennsylvania, United States) and cyantraniliprole (Verimark; FMC Corporation, Philadelphia, Pennsylvania, United States), and 1 control on EAB larval mortality and parasitism (Table 1). The low cyantraniliprole rate treatment (cya_low) also included the fungicide azoxystrobin as an ingredient, and the high-rate cyantraniliprole treatment (cya_high) included liquid fertilizer (Table 1). Because we did not test these compounds separately, it is unclear how they may have influenced EAB larvae or biological control rates separately from cyantraniliprole. The nearest parasitoid releases reported in the Map Biocontrol database (https://www.mapbiocontrol.org/home/maps/, accessed 27 January 2026), occurred in September 2017 at Raven Run Nature Sanctuary (37.895383, −84.390183) where 2,275 female *Tetrastichus planipennisi* and 200 *Oobius agrili* were released, at Burn Bridge (37.432612, −84.174869) where 804 *T. planipennisi* were released, and at Central Kentucky Wildlife Refuge (37.324051, −84.545330) where 804 *T. planipennisi* were released. Raven Run is 21.5 km away from TFEA, Burn Bridge is 33.2 km away, and Central Kentucky Wildlife Refuge is 48.7 km away. The nearest locations where *Spathius agrili* had been released were both in Lexington, Kentucky. On 20 May 2013 in River Hill Park (37.969187, −84.486194), 750 *Spathius* sp. and 1,135 *T. planipennisi* were released and on 16 October 2012, 730 *Spathius* sp. and 974 *T. planipennisi* were released in Shillito Park (37.984460, −84.537040). These 2 parks are 32.8 km and 36.6 km away from TFEA, respectively. *Spathius galinae* was not released in Kentucky until 2025 and no releases occurred in counties bordering Madison County where TFEA is located. No parasitoid releases have ever occurred in Madison County.

**Table 1.** Active ingredients and rates used for each treatment as well as average EAB densities and woodpecker attack marks recorded on a per tree basis within each treatment

Treatment	Active ingredient	Rate (mL product per L of water) per cm of DBH	Rate (g of a.i. per L of water per cm DBH)	Average ± SE DBH in cm	Average ± SE debarked surface area in m^2^ per tree	Average ± SE living EAB density per m^2^	Average ± SE woodpecker predated larvae per m^2^	Average ± SE dead EAB density per m^2^	Average ± SE percent parasitism
**Chl_low**	Chlorantraniliprole	0.083	0.040	14.2 ± 2.9	0.940 ± 0.134	13.7 ± 6.3	17.9 ± 9.5	4.8 ± 2.1	13.9 ± 4.6
**Chl_med**	Chlorantraniliprole	0.166	0.079	9.7 ± 0.9	0.916 ± 0.087	5.6 ± 3.1	8.0 ± 4.4	3.9 ± 1.9	42.9 ± 16.5
**Chl_high**	Chlorantraniliprole	0.329	0.157	8.0 ± 0.8	0.918 ± 0.064	1.3 ± 0.5	3.3 ± 1.5	1.1 ± 0.3	36.7 ± 12.2
**Cya_low**	Cyantraniliprole	0.384	0.072	10.0 ± 2.9	0.791 ± 0.102	6.7 ± 3.0	6.9 ± 3.6	2.9 ± 1.9	11.5 ± 6.4
**Cya_med**	Cyantraniliprole	0.769	0.143	12.6 ± 1.8	0.852 ± 0.072	4.7 ± 2.0	7.3 ± 3.4	3.8 ± 1.7	34.1 ± 2.3
**Cya_high**	Cyantraniliprole	1.54	0.287	11.7 ± 2.1	0.915 ± 0.064	5.7 ± 3.0	10.1 ± 5.2	3.8 ± 1.9	24.4 ± 12.5
**Control**	NA	NA	NA	11.0 ± 1.3	1.069 ± 0.114	9.5 ± 4.2	13.3 ± 7.0	3.9 ± 2.3	36.4 ± 13.7

All treatments were applied as a drench in 3.8 L of water. Averages are reported on a per square meter basis using corrected surface area values after accounting for dead phloem. Treatment codes as listed in Table 2. Trade names for the chlorantraniliprole and cyantraniliprole formulations were Durentis and Verimark, respectively (both FMC Corporation, Philadelphia, Pennsylvania, United States). The Cya_low treatment also included the fungicide azoxystrobin as an ingredient, and the treatment Cya_high included liquid fertilizer.

We selected trees with signs of EAB infestation (healed over galleries, recent attack marks from woodpeckers, noticeable canopy dieback, or D-shaped exit holes). The mean ± SE DBH of all trees was 11.0 ± 0.7 cm (range = 25.7 to 5.8 cm). Nine trees were multistemmed, so DBH values for each stem were combined when recording DBH for these trees. We used a random number generator to assign each tree to 1 of the 7 treatments in a completely randomized design so that there was a total of 6 trees in each treatment. Rates for each product are listed in Table 1. All products were mixed into 3.8 L of water and applied at the base of trees, after raking away leaf debris, as a drench application on 21 August 2024. We used this volume of water to ensure sufficient contact of each product with tree root zones so that uptake was maximized. Light rainfall (<2.5 cm total) had occurred daily from 16 to 19 August in Richmond, Kentucky (Richmond 8E weather station: https://weather.uky.edu/php/kypmsites_list.php, accessed 27 January 2025) so some soil moisture likely remained at the site on the day of application.

On 17 March 2025, we felled and dissected trees to record the abundance of living and dead EAB larvae and pupae as well as parasitism rates. For multistemmed trees, we only felled 1 stem (>5 cm DBH) for dissections. We cut trees into 1 m sections and removed the bottom 4 m for dissection. Thus, four 1 m sections were collected from each tree. For certain trees (2 trees for chl_low treatment, and 1 tree for chl_med and cya_low treatments, Table 1), we only collected data from 3 sections either because 1 section was entirely dead, was lost in transit to the lab, or was <2.5 cm diameter (a size below which EAB typically do not infest trees) (Cappaert et al. 2005). All sections were brought back to the lab and stored in a cold room until dissection. We measured the length and diameter of each section to calculate curved surface area. Surface area estimates were modified by the proportion of dead phloem on each dissected section based on visual estimates. One tree receiving the chl_high treatment was dead at the time of felling and removed from analysis. Woodpecker predation on EAB over the winter of 2024 to 2025 was quantified by counting the number of recent woodpecker feeding holes based on the color of wood where bark had been removed (Supplementary Fig. S1). We removed bark with drawknives and recorded the number of all living and dead EAB larvae or pupae. Larvae were recorded based on their developmental stages (first instar [L1], second instar [L2], third instar [L3], fourth instar [L4], J-larvae or prepupae). We did not distinguish between J-larvae and prepupae. We also counted all EAB galleries that had developing parasitoid life stages within them and took photos for reference. We did not record the number of immature parasitoid life stages within individual galleries.

A subset of all immature parasitoids were removed from trees (38 out of 87 total parasitized galleries from 13 trees), placed in petri dishes and reared in an incubator at 25 °C with a 12:12 h (L:D) photoperiod to allow adults to emerge. We used photos of immature parasitoids that were reared to adults to aid in identifying immature parasitoids that were not reared. All living EAB larvae that were not damaged when removed from log sections were inspected for internal parasitism by *P. sulcata*. This was done by severing the last larval segment near the base of the urogomphi, squeezing out the body contents, and inspecting the contents for parasitoid larvae using a stereo microscope.

Kruskal–Wallis tests were used to determine if the DBH across trees and the surface area debarked, differed across treatments. We fit generalized linear models (GLMs) to determine if living EAB density, dead EAB density, woodpecker predation density, density of parasitized EAB larvae (recorded while debarking trees), and total parasitism per square meter (combining *P. sulcata* counts from dissected larvae with in situ parasitized larvae) were influenced by treatment. We also fit GLMs to test if living and dead early instar larvae (L1 and L2) were influenced by treatment relative to the control, and we fit similar models for living and dead late development stages (L3, L4, J-larvae/prepupae, pupae). These models were fit with the “glmmTMB” package (McGillycuddy et al. 2025) using a negative binomial distribution (log-link function) and each included an offset term to account for the surface area over which counts were recorded (modified based on the percentage of dead phloem). We used the negative binomial distribution for these models due to evidence of overdispersion in model residuals. A penalized binomial GLM (with logit transformation) using the “brglm” package (Kosmidis and Firth 2021) was used to determine if the proportion of larvae parasitized by *P. sulcata* was influenced by treatment. This approach was used to address potential bias introduced by the small number of larvae dissected in certain treatments and produced more interpretable model coefficients. We fit 1 binomial GLMM to determine if treatment influenced the proportion of larvae parasitized by *T. planipennisi* and while a second model tested effects of treatment on parasitism by *S. galinae* and *Atanycolus* spp. together. Both models included an observation level random effect term to address overdispersion (Harrison 2015). We modeled the effect of treatment on *T. planipennisi* separately from *S. galinae* and *Atanycolus* spp. because *T. planipennisi* is an endoparasitoid, while the latter 2 species are ectoparasitoids (Bauer et al. 2015) and thus may be exposed to differing levels of pesticide within treated trees. When calculating the proportion of parasitized larvae by these species, first and second instar EABs were excluded as all 3 parasitoid species predominantly parasitize third and fourth instars (Liu et al. 2007, Cappaert and McCullough 2009, Belokobylskij et al. 2012). We also used a binomial GLM to determine how treatment influenced overall EAB mortality when all sources of mortality (dead, predated, and parasitized larvae) were accounted for. Significance was evaluated with Wald tests based on comparisons between each treatment and the control. We evaluated the fit of models using the “DHARMa” package (Hartig 2025). We fit a GLMM (negative binomial, log-link function) to determine if treatment interacted with log section to influence the density of living larvae as it was suspected greater mortality might occur lower in the trunk if diamides have limited translocation ability within trees. This model included a random intercept term for each tree’s ID to account for the repeated measures data and an offset term for log surface area. Likelihood ratio tests were used to assess significance of predictors in this model.

## Results

**Fig. 1.**
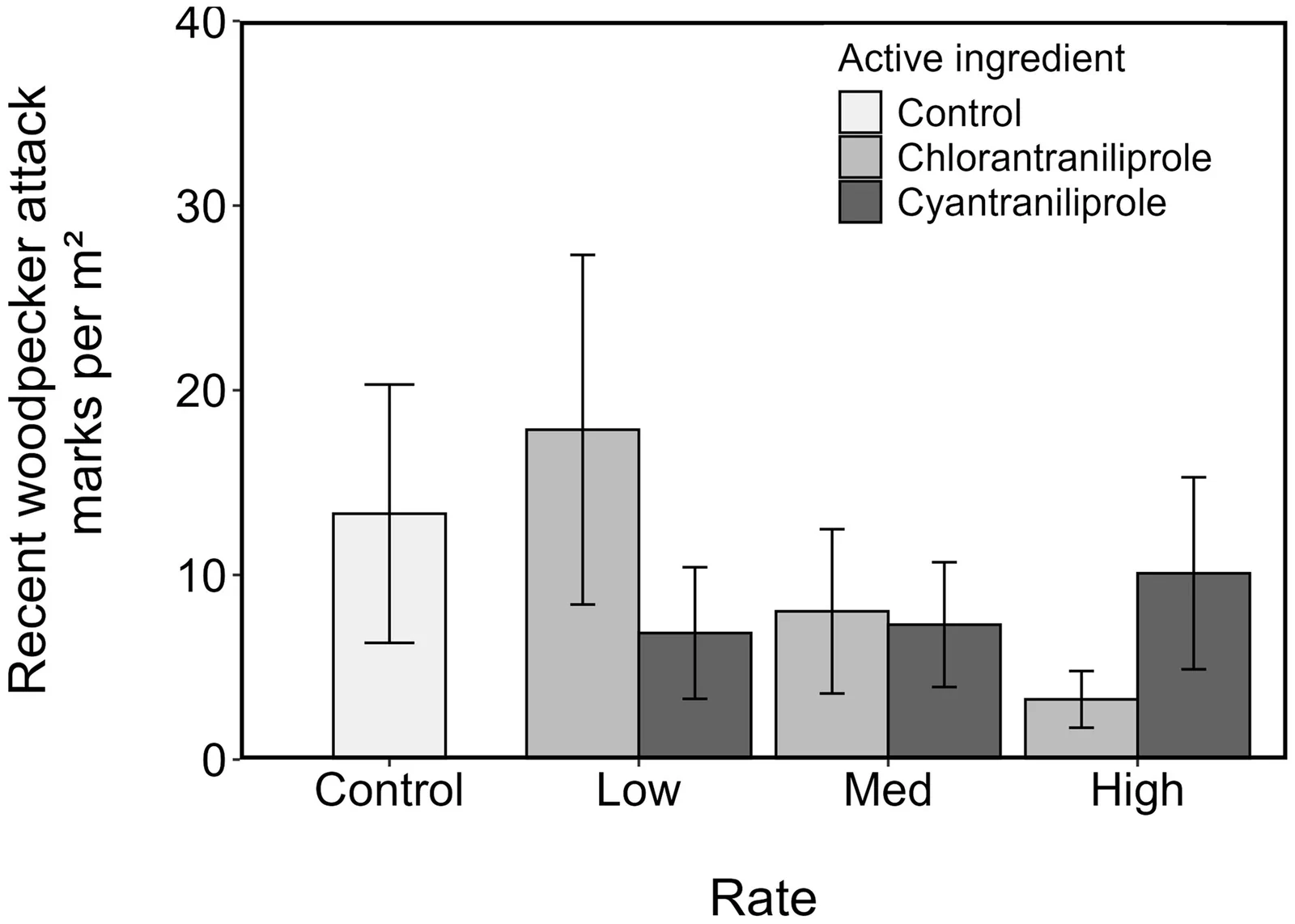
Effect of soil drench with 2 diamide insecticides on the density of larvae recently predated by woodpeckers. There were no significant differences for either active ingredient at any rate relative to the control treatment (Table 2).

**Fig. 2.**
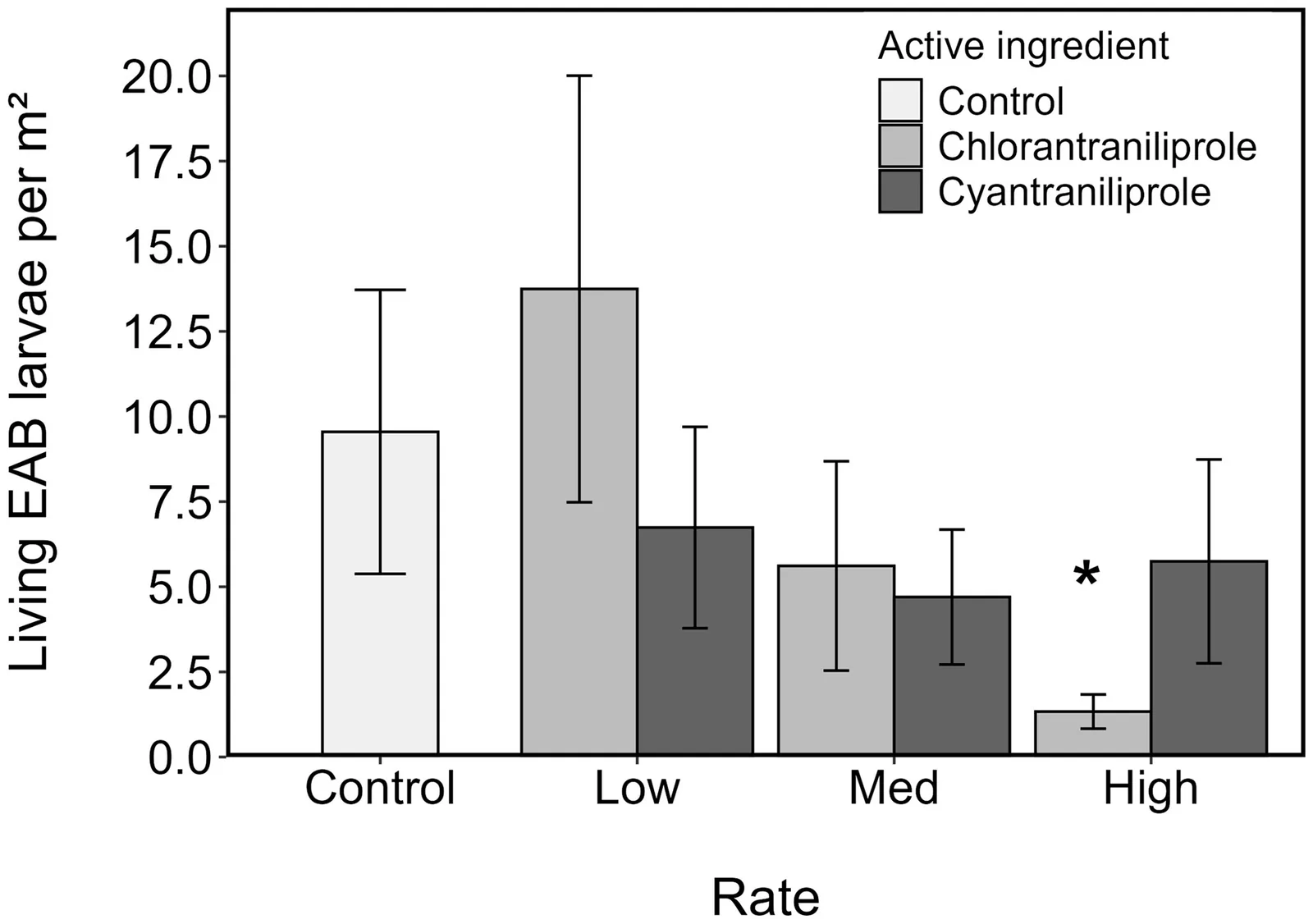
Effect of treatment on living EAB densities (total larvae and pupae) per square meter (Table 2). Exact rates used for each active ingredient are listed in Table 1. Asterisk indicates significant differences of a treatment relative to the control. Only the high-rate chlorantraniliprole treatment had significantly fewer living larvae per m^2^ relative to the control treatment (β = −1.975 ± 0.834, *Z* = −2.367, *P *= 0.018).

We debarked a total of 164 meter-length logs (mean ± SE diameter = 8.0 ± 0.2), which were used for statistical analysis, representing a total of 40.9 m^2^ of surface area (corrected to 37.5 m^2^ after accounting for dead phloem). Average diameter for all dissected logs, starting from the base of trees, was 9.6 ± 0.3 cm for meter one, 8.6 ± 3.1 cm for meter two, 7.2 ± 0.3 cm for meter three, and 6.3 ± 0.3 cm for meter four. Total tree DBH was not significantly different across treatments (χ^2^ = 2.32, df = 6, *P *= 0.89), nor was total debarked surface area (χ^2^ = 4.01, df = 6, *P *= 0.68) or corrected surface area after accounting for dead phloem (χ^2^ = 3.65, df = 6, *P *= 0.73). We recorded a total of 245 living EAB larvae, 125 dead larvae, 9 living pupae, 6 dead pupae, 343 predated larvae, and 87 immature parasitoids. Total living and dead EAB across each development stage are listed in Supplementary Table S1. The percentage of larvae recently predated by woodpeckers averaged 37.9 ± 3.4% across all trees and ranged from 30.0 ± 8.6% in the medium rate chlorantraniliprole treatment (chl_med) to 44.0 ± 14.2% in the low-rate cyantraniliprole treatment (cya_low) (Fig. 1, Table 2). Only the high chlorantraniliprole rate (chl_high) resulted in significantly fewer living larvae and pupae (1.3 ± 0.5 EAB per m^2^) relative to the control (9.5 ± 4.2 EAB per m^2^) (Fig. 2, Table 2). Density of living early instar larvae (L1 and L2) was higher in the low chlorantraniliprole rate (chl_low) (3.2 ± 2.9 EAB per m^2^) relative to the control (0.1 ± 0.1 EAB per m^2^), and density of living late development stages (L3, L4, J-larvae/prepupae, and pupae) were lower in the high chlorantraniliprole rate (chl_high) (1.3 ± 0.5 EAB per m^2^) relative to the control (9.8 ± 4.1 EAB per m^2^). Density of dead early instars was significantly higher in the low chlorantraniliprole rate (chl_low) (2.2 ± 1.5 EAB per m^2^) relative to the control (0.2 ± 0.2 EAB per m^2^), while no treatment differed in the density of dead, late development stages relative to the control (Supplementary Table S2).

**Table 2.** Wald tests comparing each insecticide treatment to the control

Response	Treatment	Estimate	SE	*Z*	*P*
**Living EAB per m^2^**	Intercept (Control)	2.257	0.499	4.523	<0.001
	Chl_low	0.352	0.706	0.498	0.618
	Chl_med	−0.559	0.722	−0.773	0.439
	Chl_high	−**1.975**	**0.834**	−**2.367**	**0.018**
	Cya_low	−0.318	0.712	−0.446	0.656
	Cya_med	−0.723	0.726	−0.995	0.320
	Cya_high	−0.492	0.714	−0.689	0.491
**Dead EAB per m^2^**	Intercept (Control)	1.381	0.531	2.600	<0.001
	Chl_low	0.155	0.759	0.204	0.839
	Chl_med	−0.053	0.762	−0.069	0.945
	Chl_high	−1.315	0.881	−1.492	0.136
	Cya_low	−0.262	0.764	−0.343	0.731
	Cya_med	−0.077	0.765	−0.100	0.920
	Cya_high	−0.036	0.755	−0.048	0.961
**Living early instar EAB per m^2^**	Intercept (Control)	−1.952	1.191	−1.638	0.101
	Chl_low	**3.065**	**1.379**	**2.223**	**0.026**
	Chl_med	1.511	1.473	1.026	0.305
	Chl_high	−18.650	13,895.507	−0.001	0.999
	Cya_low	2.027	1.413	1.435	0.151
	Cya_med	1.060	1.525	0.695	0.488
	Cya_high	1.591	1.439	1.105	0.269
**Dead early instar EAB per m^2^**	Intercept (Control)	−1.841	1.069	−1.722	0.850
	Chl_low	**2.558**	**1.177**	**2.173**	**0.030**
	Chl_med	0.820	1.34	0.614	0.539
	Chl_high	0.302	1.520	0.199	0.843
	Cya_low	1.787	1.220	1.464	0.143
	Cya_med	2.189	1.193	1.834	0.067
	Cya_high	1.895	1.204	1.574	0.115
**Living late stage EAB per m^2^**	Intercept (Control)	2.285	0.510	4.476	<0.001
	Chl_low	0.083	0.725	0.115	0.909
	Chl_med	−0.271	0.733	−0.369	0.712
	Chl_high	−**2.003**	**0.850**	−**2.357**	**0.018**
	Cya_low	−0.502	0.732	−0.686	0.493
	Cya_med	−0.847	0.745	−1.136	0.256
	Cya_high	−0.642	0.732	−0.877	0.381
**Dead late stage EAB per m^2^**	Intercept (Control)	1.335	0.606	2.204	0.028
	Chl_low	−0.443	0.888	−0.498	0.618
	Chl_med	−0.098	0.870	−0.113	0.910
	Chl_high	−1.480	1.006	−1.471	0.141
	Cya_low	−0.470	0.877	−0.535	0.593
	Cya_med	−0.532	0.892	−0.596	0.551
	Cya_high	−0.300	0.868	−0.345	0.730
**Woodpecker predated larvae per m^2^**	Intercept (Control)	2.588	0.554	4.676	<0.001
	Chl_low	0.286	0.784	0.365	0.715
	Chl_med	−0.521	0.793	−0.657	0.511
	Chl_high	−1.416	0.855	−1.656	0.098
	Cya_low	−0.650	0.793	−0.819	0.413
	Cya_med	−0.609	0.794	−0.766	0.444
	Cya_high	−0.271	0.785	−0.344	0.731
**Immature parasitoids per m^2^**	Intercept (Control)	0.495	0.620	0.797	0.425
	Chl_low	0.419	0.874	0.480	0.631
	Chl_med	0.521	0.861	0.606	0.545
	Chl_high	0.032	0.928	0.034	0.973
	Cya_low	0.025	0.884	0.028	0.977
	Cya_med	0.943	0.853	1.106	0.269
	Cya_high	1.016	0.848	1.197	0.231
**Total combined parasitism per m^2^**	Intercept (Control)	**1.634**	**0.575**	**2.842**	**0.004**
	Chl_low	−0.134	0.826	−0.162	0.871
	Chl_med	−0.236	0.823	−0.287	0.774
	Chl_high	−1.108	0.904	−1.226	0.220
	Cya_low	−0.455	0.826	−0.551	0.582
	Cya_med	−0.140	0.824	−0.170	0.865
	Cya_high	0.010	0.816	0.010	0.992
**Proportion total larval mortality**	Intercept (Control)	0.850	0.152	5.599	<0.001
	Chl_low	−0.215	0.218	−0.982	0.326
	Chl_med	0.249	0.273	0.913	0.361
	Chl_high	0.655	0.746	1.374	0.169
	Cya_low	−0.208	0.257	−0.840	0.401
	Cya_med	0.322	0.287	1.121	0.262
	Cya_high	0.285	0.244	1.168	0.243
**Proportion of larvae parasitized by *P. sulcata***	Intercept (Control)	−0.355	0.272	−1.306	0.192
	Chl_low	−**1.387**	**0.463**	−**2.995**	**0.003**
	Chl_med	−0.794	0.533	−1.490	0.136
	Chl_high	−2.043	1.641	−1.245	0.213
	Cya_low	−0.608	0.457	−1.331	0.183
	Cya_med	−**2.260**	**0.907**	−**2.491**	**0.013**
	Cya_high	−**1.456**	**0.584**	−**2.494**	**0.013**
**Proportion of larvae parasitized by *S. galinae* and *Atanycolus* spp.**	Intercept (Control)	−2.407	0.538	−4.478	<0.001
	Chl_low	0.058	0.729	0.079	0.937
	Chl_med	1.308	0.696	1.878	0.060
	Chl_high	0.501	0.965	0.519	0.604
	Cya_low	0.739	0.719	1.028	0.304
	Cya_med	**1.583**	**0.688**	**2.301**	**0.021**
	Cya_high	0.748	0.696	1.075	0.283
**Proportion of larvae parasitized by *T. planipennisi***	Intercept (Control)	−3.749	1.025	−3.658	<0.001
	Chl_low	−0.028	1.468	−0.019	0.985
	Chl_med	0.270	1.521	0.178	0.859
	Chl_high	2.524	1.388	1.819	0.069
	Cya_low	−0.808	1.753	−0.461	0.645
	Cya_med	1.675	1.356	1.235	0.217
	Cya_high	2.243	1.336	1.679	0.093

The control treatment was set as the reference level for these tests. Treatment codes refer to those listed in Table 1.

**Fig. 3.**
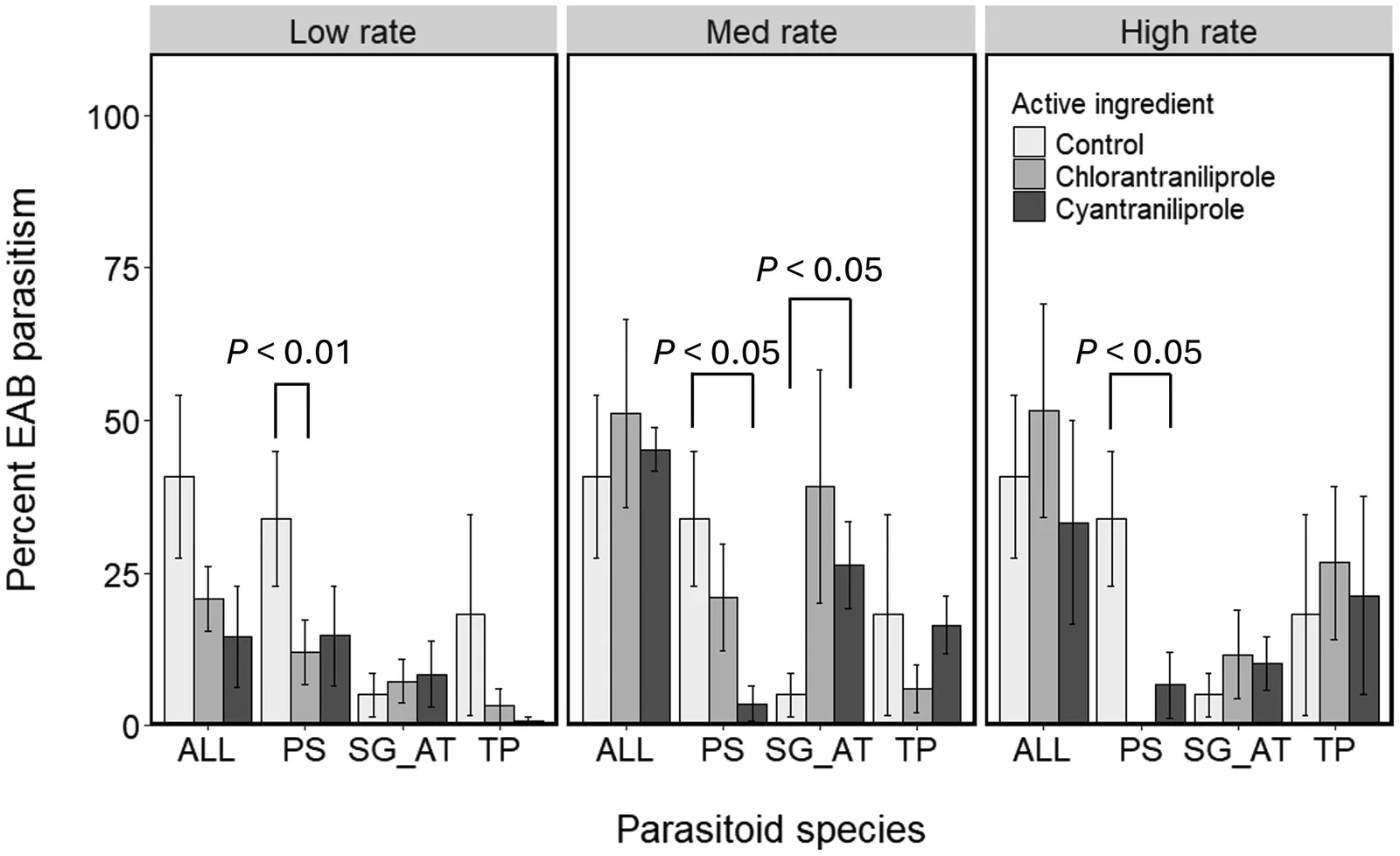
Effect of treatment on parasitism rates by different wasp species. Note that the control bars in each facet (low rate, med rate, and high rate) are the same, but are repeated for comparison with each active ingredient at each rate. Test statistics and *P*-values for all comparisons are listed in Table 2. Parasitism averages for each species across all treatments are listed in Table 3. Abbreviations: ALL = all parasitoid species; PS = *P. sulcata*; SG_AT = *S. galinae* and *Atanycolus* spp.; TP = *T. planipennisi*.

**Fig. 4.**
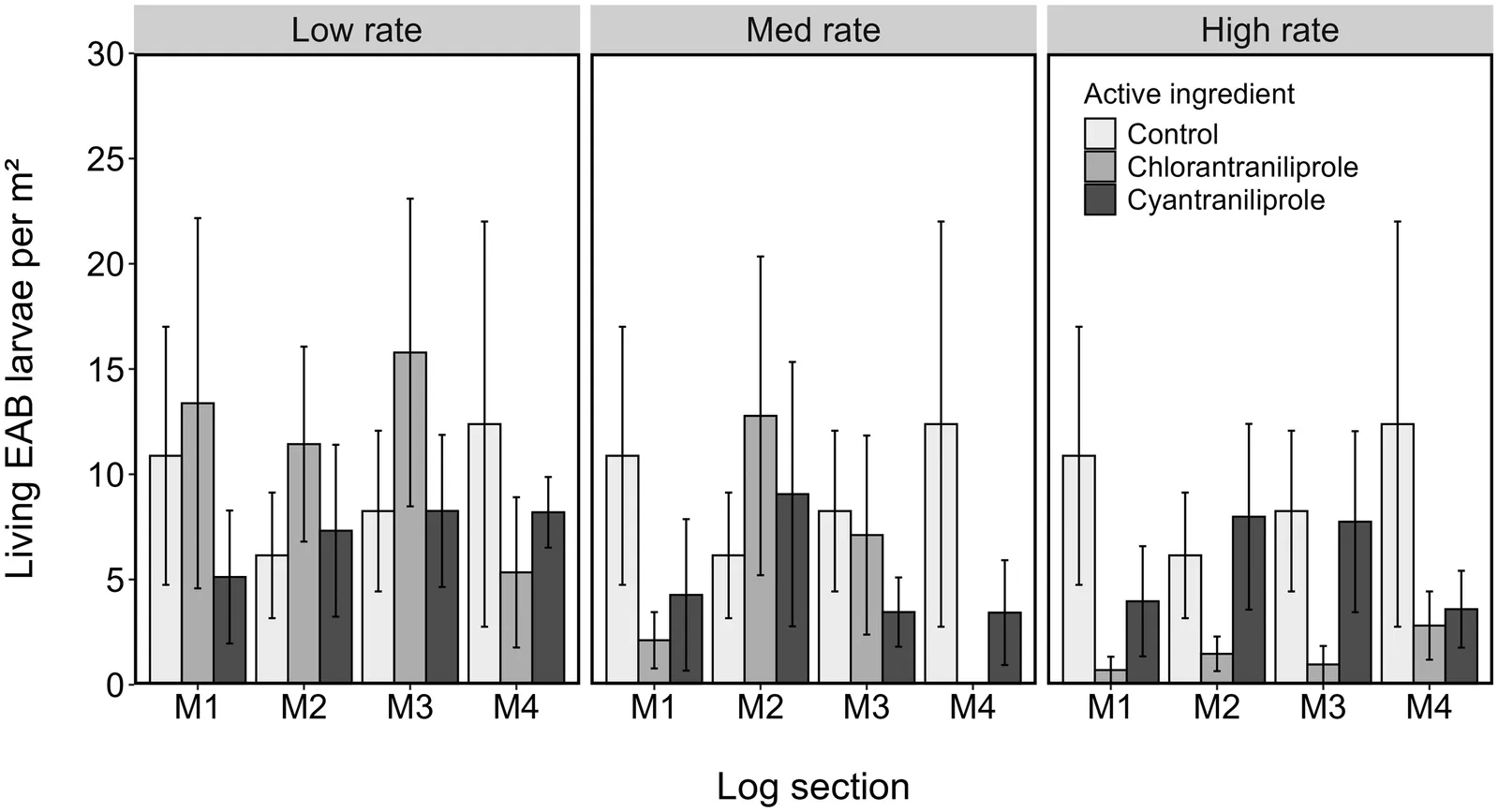
Effect of treatment on the average density of living larvae and pupae found within each meter section of each treatment. Control bars are repeated in each facet (low rate, med rate, and high rate) to enable comparison with active ingredients at each rate. Treatments did not interact with log section to influence the number of living larvae (χ^2^ = 15.78, df = 18, *P *= 0.61) and both main effects were nonsignificant (treatment: χ^2^ = 6.13, df = 6, *P *= 0.41; log section: χ^2^ = 5.17, df = 3, *P *= 0.16).

**Fig. 5.**
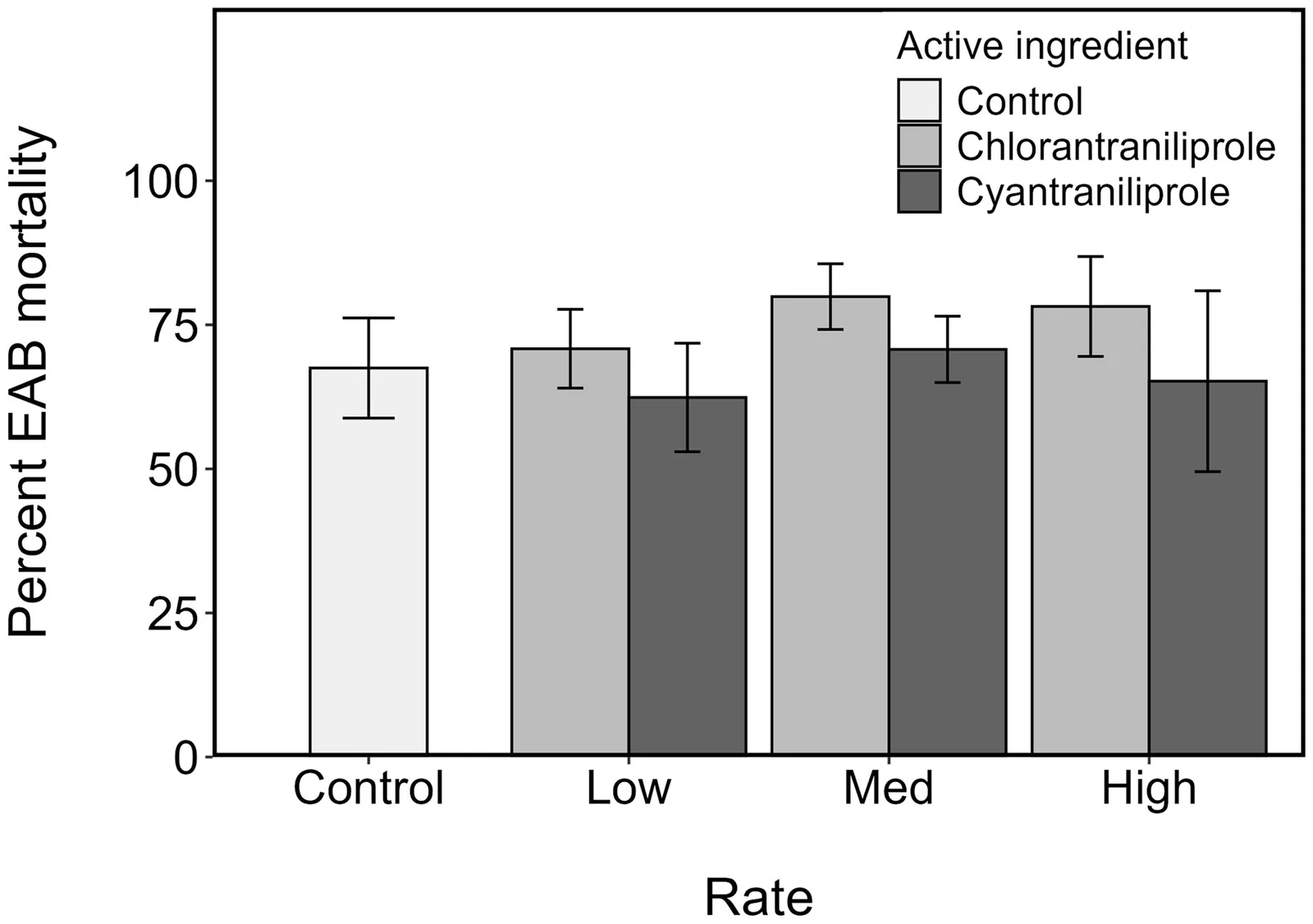
Percentage of EAB larval mortality combined for all sources across all treatments. Mortality was calculated by dividing the total number of parasitized larvae, dead larvae, and recent woodpecker attack marks by the number of woodpecker marks and living, dead, and parasitized larvae. Trees where no larvae, no woodpecker marks, and no parasitoids were found are excluded (*n* = 5). Percent EAB mortality did not differ between any treatment and the control (Table 2).

No treatment had significantly different dead EAB densities, woodpecker predation, in situ EAB parasitism density (all species except *P. sulcata*), or total combined parasitized larval densities (all species together) relative to the control (Table 2). However, the proportion of larvae parasitized by *P. sulcata* was significantly lower than the control (33.9 ± 11.1%) in the chl_low (11.9 ± 5.3%), cya_med (3.6 ± 2.9%), and cya_high (6.7 ± 5.4%) treatments (Fig. 3, Table 2). We only dissected 5 larvae across all trees with the chl_high treatment (due to few larvae found in these trees) and did not detect any internal parasitism. The proportion of larvae parasitized by external parasitoids—*Atanycolus* spp. and *S. galinae—*in the cya_med treatment (26.2 ± 7.1%) was significantly higher than the control (5.1 ± 3.6%) (Table 2, Fig. 3). The proportion of larvae parasitized by *T. planipennisi* did not differ between any treatment and the control (Table 2, Fig. 3). Treatments did not interact with log section to influence the number of living larvae (χ^2^ = 15.78, df = 18, *P *= 0.61) and both main effects were nonsignificant (treatment: χ^2^ = 6.13, df = 6, *P *= 0.41; log section: χ^2^ = 5.17, df = 3, *P *= 0.16, Fig. 4). When all sources of EAB mortality are combined (dead larvae, woodpecker predation, internal and external parasitism) overall mortality averaged 70.1 ± 3.4% across all treatments and ranged from 65.2 ± 15.7% in the high cyantraniliprole treatment (cya_high) to 79.9 ± 8.7% in the medium rate chlorantraniliprole treatment (chl_med), but no treatment differed significantly from the control (Fig. 5, Table 2).

In situ larval parasitism by *Spathius* spp., *T. planipennisi*, *Atanycolus* spp, and unknown wasps, recorded when debarking trees, averaged 30.9 ± 5.1% across all treatments and ranged from 9.2 ± 5.9% for the cya_low treatment to 46.2 ± 4.1% for the cya_med treatment (Table 3). When parasitism rates from *P. sulcata* are included, an average of 36.3 ± 4.8% of EAB larvae were parasitized across all treatments, ranging from 14.5 ± 8.3% for the cya_low treatment to 51.7 ± 17.6% for the chl_high treatment (Table 3, Fig. 5). Parasitism by the 2 introduced species, *T. planipennisi* and *S. galinae*, averaged 21.4 ± 4.6% across all treatments and ranged from 3.7 ± 3.4% in the cyan_low treatment to 38.9 ± 17.2% in the chl_med treatment (Table 3). We dissected a total of 232 EAB larvae, of which 22.4% were parasitized by *P. sulcata*. Across all trees where larvae were dissected, an average of 13.5 ± 3.5% of larvae were parasitized by *P. sulcata*, and parasitism rates were highest for the control treatment (33.9 ± 11.1%) and lowest for chl_high at 0% (Table 3). Parasitism rates for each species within each treatment are listed in Table 3. Out of all 87 parasitized larvae recorded when debarking, 38% were parasitized by *S. galinae*, 37% by *T. planipennisi*, 33% by *Atanycolus* spp., and 8% were unknown. We reared 38 immature parasitoids (collected from 22 galleries) in an incubator until adult wasps emerged, and 14 wasps (collected from 10 galleries) were identified as *S. galinae*, 14 wasps (recorded from 2 galleries) were identified as *T. planipennisi*, 9 wasps (collected from 9 galleries) were identified as *Atanycolus* spp., and 1 wasp from 1 gallery was unknown but could be *Eupelmas* sp. (Hymenoptera: Eupelmidae).

**Table 3.** EAB parasitism rates for all wasp species across all treatments

	Parasitoid species (% of total)						
Treatment	*T. planipennisi*	*S. galinae*	*T. planipennisi* and *S. galinae* combined	*Atanycolus* spp.	*P. sulcata*	Unknown	All species
**chl_low**	3.2 ± 2.9	2.9 ± 2.6	6.1 ± 3.4	4.4 ± 1.6	11.9 ± 5.3	4.7 ± 3.0	20.9 ± 5.3
**chl_med**	6.1 ± 3.9	32.8 ± 18.9	38.9 ± 17.1	6.5 ± 5.3	20.9 ± 8.7	0	51.2 ± 15.4
**chl_high**	26.7 ± 12.5	0	26.7 ± 12.5	11.7 ± 7.3	0	13.3 ± 8.2	51.7 ± 17.6
**cya_low**	0.7 ± 0.7	3.0 ± 2.7	3.7 ± 3.4	5.5 ± 3.1	14.7 ± 8.1	0	14.5 ± 8.3
**cya_med**	16.5 ± 4.7	14.5 ± 5.4	31.0 ± 4.5	11.7 ± 5.0	3.6 ± 2.9	3.6 ± 1.7	45.3 ± 3.6
**cya_high**	21.3 ± 16.3	5.5 ± 2.1	26.8 ± 17.2	4.7 ± 2.7	6.7 ± 5.4	0	33.3 ± 16.7
**Control**	18.2 ± 16.4	3.5 ± 3.5	21.7 ± 16.0	1.5 ± 1.5	33.9 ± 11.1	1.5 ± 1.5	40.8 ± 13.4
**All treatments combined**	22.0 ± 10.1	7.8 ± 2.9	21.3 ± 4.6	6.3 ± 1.5	13.5 ± 3.0	3.3 ± 1.3	36.3 ± 41.8

Parasitism rates for *T. planipennisi, S. galinae, Atanycolus* spp., and unknown wasps exclude L1 and L2 larvae as these stages are rarely targeted by these species. Parasitism rates for *P. sulcata* include all EAB larval stages and are calculated based on all 232 dissected larvae.

## Discussion

Releases of parasitoid wasps and insecticide treatments remain effective EAB management strategies but studies on their combined impact on overall EAB population reduction are limited. Only the high rate of chlorantraniliprole significantly reduced live larval densities relative to controls, while no rate of cyantraniliprole influenced larval densities. We found similar EAB parasitism rates by *P. sulcata, Atanycolus* spp. and Asian species as has been recorded in Connecticut, Michigan, Maryland, New York, and Ontario (Duan et al. 2015, Roscoe et al. 2016, Aker et al. 2022, Quinn et al. 2022a, 2022b, Duan et al. 2023b, Morris et al. 2023, Wilson et al. 2024). *Tetrastichus planipennisi* parasitized 22% of larvae even though this species was last released 7 years before this study and >20 km away. *Spathius galinae* parasitized 8% of larvae even though it had not been released in Kentucky at the time of this study. Our data suggest these 2 parasitoids species may have successfully established at our field site where they were a significant source of EAB larval mortality. However, because we do not have data across multiple years or data from other sites in central Kentucky, the full extent of dispersal and establishment by each species is not clear. Woodpeckers are often recorded killing a higher percentage of EAB larvae relative to parasitoids (Lindell et al. 2008, Jennings et al. 2013, Duan et al. 2015, Wilson et al. 2024), but in this study, predation rates (38%) were similar to combined parasitism rates by native and introduced species (36%). Biological control by *P. sulcata*, but not by woodpeckers or any other wasp species, was reduced by at least 1 treatment for each tested active ingredient. While diamides can be used in conjunction with releases of introduced parasitoids, they may reduce parasitism by *P. sulcata* in treated trees.

The failure of certain insecticide treatments to control EAB might be attributed to limited translocation potential within treated trees and treatment timing. EAB management guidelines suggest treating when dieback is 30% or less as heavily damaged trees have compromised vascular structure which hinders insecticide translocation (Sadof et al. 2023). Dieback was not measured in this study, thus phloem and xylem damage may have limited insecticide translocation in certain trees, although we do not know this for certain (Cappaert et al. 2005, McCullough 2020).

When insecticides were applied in August, most EAB larvae were likely third or fourth instars, based on studies from Virginia (Ragozzino et al. 2020) and North Carolina (Bohannon et al. 2022). A greater proportion of these stages tend to survive insecticide treatments relative to first and second instars (e.g. McCullough et al. 2011). Since these stages are more resistant to insecticides, the timing of our applications may have limited efficacy of some treatments. Presence of both first and second instars when trees were debarked in March (16% of all larvae recorded) suggests some early instars were present at the time of treatment and may have been killed by the insecticides. These small larvae can die when tunneling into treated trees after hatching and are often destroyed while debarking trees. This effect likely explains significant differences in living larval densities in other EAB insecticide studies despite many late instar larvae remaining alive (Smitley et al. 2010, McCullough et al. 2011, 2019). In our study, only the high-rate chlorantraniliprole treatment had significantly fewer late development stages (L3, L4, J-larvae/prepupae, and pupae) relative to controls. This may indicate that the treatment killed early instar larvae present within trees in late summer, resulting in fewer late stage larvae and pupae in spring, and/or that higher rates of chlorantraniliprole are needed to produce mortality in late development stages when applied in late summer.

Previous studies found that systemic insecticides were most effective on EAB when applied in spring rather than in autumn (Smitley et al. 2015, Bick et al. 2018). These differences were attributed to increased translocation of active ingredients before EAB larval feeding damage impaired tree vascular structure. We applied insecticides in late August which is better described as late summer rather than autumn in central Kentucky. Daily average temperatures were 22.9 °C in Madison County in August 2024 and average high and low temperatures for the month were 29.5 °C and 16.3 °C, respectively (Kentucky Mesonet 2024). Transpiration, and thus xylem uptake of water, would still have been active at this point as leaf senescence would not begin for another 1 to 2 months after application timing. Smitley et al. (2015) found that while spring applications generally resulted in better EAB control than fall applications, ash treated with a high rate of imidacloprid in October and November had healthier canopies relative to controls in the following year in 1 experiment, and that canopy dieback in fall-treated trees was similar to spring-treated trees. However, fall-treated trees at a lower rate had worse dieback compared to spring-treated trees at the same rate. We applied insecticides 2 to 3 months earlier than Smitley et al. (2015) and tested a different pesticide class. Thus, while our application timing may not have aligned with recommendations, there is not sufficient evidence that timing alone would have prevented insecticides from being absorbed by trees. October applications of chlorantraniliprole (2 months after our timing) as a drench to roses has been shown to reduce roseslug sawfly damage in the following April, as well as Japanese beetle herbivory in the following June, in central Kentucky (Redmond and Potter 2017). Often ash dieback from EAB is not noticed until late summer and our application timing may reflect when homeowners, arborists, or foresters notice a new infestation and are able to apply treatments (Herms et al. 2014). While this timing may not be ideal, our results suggest such applications could limit further damage to infested trees.

Pesticide residues were not measured in this study, so it is unknown where diamides translocated following application. Dinotefuran and imidacloprid become concentrated at application sites (lower trunk or roots), as well as within leaves, and may therefore kill more EAB adults relative to larvae feeding on treated trees (Mota-Sanchez et al. 2009, Olson et al. 2020). As EAB larvae feed on phloem and cambium tissue, they score the sapwood which allows xylem-mobile insecticides to contact larvae (Cappaert et al. 2005, McCullough 2020). Both chlorantraniliprole and cyantraniliprole are translocated through xylem and cyantraniliprole has greater solubility between the 2, and thus greater translocation potential (Selby et al. 2017, Lahm et al. 2019). However, only chlorantraniliprole significantly reduced larval densities 6 months after treatment, suggesting that solubility alone does not explain the observed differences in efficacy between the insecticides. The high-rate chlorantraniliprole treatment had the lowest larval densities across all treatments which could result from low infestation pressure in selected trees prior to treatment. While we did not collect branch samples to quantify pretreatment EAB densities, it is not clear if such samples would accurately predict densities found in the trunk prior to applying treatments. We specifically chose trees with visible signs of infestation for this project and randomly assigned treatments to all selected trees. We consider it more likely that low larval densities in the high-rate chlorantraniliprole treatment are due to mortality from the insecticide.

The few published field studies in which diamides provided 1 to 2 years of protection from tree pests used seedling-sized trees (Moore et al. 2021, McCarty et al. 2023), whereas studies on mature fruit trees treated with diamide drenches or sprays only found significant protection for 1 to 5 weeks (Sial and Brunner 2010, Tiwari and Stelinski 2013). In the present study, the amount of product applied within each treatment was altered based on tree DBH (as is common for most systemic insecticides); however, the average DBH of trees was only 11 cm. Applications may be less effective on mature overstory trees, although no study has tested this. Nonetheless, the present results indicate that soil drenches of chlorantraniliprole could be used to protect small diameter white ash in locations where neonicotinoids cannot be used, or where trees are too small for trunk injections. Confounding factors such as the unknown extent of preexisting tree vascular structure damage, or summer rather than spring application, or the relative abundance of late compared to early instar larvae within treated trees, may have contributed to cyantraniliprole not significantly reducing EAB larval densities in this study.

We also tested treatments on 6 trees per treatment which may have limited our ability to detect significant effects of certain treatments. For example, there was substantial variation living EAB densities in the low-rate chlorantraniliprole treatment across trees (mean ± SE = 13.7 ± 6.3 larvae per m^2^) which could have obscured significant effects when compared to the control which also had high variation across replicates (9.5 ± 4.2 larvae per m^2^). Nonetheless, other studies have used similar sample sizes to study effects of systemic insecticides on EAB and have been able to detect an effect of certain treatments, as we did for the high-rate chlorantraniliprole treatment (e.g. McKenzie et al. 2010, Smitley et al. 2015, Bick et al. 2017). Further research with greater replication using spring applications, at multiple rates, with multiple application methods using cyantraniliprole, chlorantraniliprole, and other diamides is warranted. Measurement of pesticide residues within leaves at set intervals following treatment would indicate the rate and extent to which diamides translocate through infested ash.

EAB parasitism by native and introduced wasps is influenced by factors including the number of individuals released, climate, release timing, availability of hosts, ash density, and bark thickness (Abell et al. 2012, Duan et al. 2018, Ragozzino et al. 2020, Quinn et al. 2022a, 2022b, Duan et al. 2023a, 2023b, Morris et al. 2024, Wilson et al. 2024). Even when releases are timed correctly and release numbers are high, parasitoid establishment can take multiple years (e.g. Duan et al. 2023b). We found high parasitism by *Spathius galinae*, even though *S. galinae* was not released in this stand and, according to the MapBiocontrol database, had not been released in Kentucky at the time of this study (MapBioControl.org 2026). Since we only reared 42% of *Spathius* spp. cocoons to adulthood, it is possible that some larvae were parasitized by native *Spathius* spp. or *S. agrili*. Substantial parasitism by *T. planipennisi* is encouraging because the nearest release of this species was over 20 km away and had last occurred 7 years prior. We found greater parasitism by *T. planipennisi* than did another study (Davidson and Rieske 2016), conducted in central Kentucky 10 years ago, indicating this species has become more established over time. Studies in New York and Connecticut found *S. galinae* dispersed at least 14 km, and *T. planipennisi* 10-14 km, within 3 to 5 years after they were released (Jones et al. 2019, Quinn et al. 2022a, 2022b). Our results suggest that introduced EAB parasitoids can disperse long distances, and parasitize 21% of EAB larvae in the upper south several years after their release, although the extent of their establishment in our region is not known. EAB has a mixture of univoltine and semivoltine lifecycles in northern latitudes, and a univoltine lifecycle at southern latitudes (Gould et al. 2020, Bohannon et al. 2022). We felled trees in spring and found that 20% of living larvae were not J-larvae, prepupae, or pupae. This indicates that the majority of EAB in central Kentucky are univoltine as has been also documented in Fayette County (Pellecchia 2020) which borders Madison County to the North, where this study was conducted. These findings explain why both *T. planipennisi* and *S. galinae*, which require susceptible EAB larvae to parasitize in late spring, appear to have established at our study site, and likely elsewhere in central Kentucky (Gould et al. 2020).

If insecticides are combined with parasitoid releases, it is important to ensure that the 2 tactics are not antagonistic. Davidson and Rieske (2016) found no negative effects of imidacloprid treatments on EAB parasitism at sites where *T. planipennisi* and *O. agrili* were released. In contrast, Morris et al. (2024), who examined the effects of emamectin benzoate trunk injections on establishment of EAB parasitoids, found that *T. planipennisi* generally established well but recoveries of *S. galinae* and *O. agrili* were low. Since parasitoids and woodpeckers will not attack dead EAB larvae, applying treatments in spring before wasps are active would presumably focus biological control agents to target untreated trees (McCullough 2020). In locations where ash is prevalent, treating even <1% of standing ash in a forest stand can result in a significant reduction in EAB densities but would leave hosts for parasitoids to target (Mercader et al. 2015). We detected reduced parasitism by *P. sulcata*, but not introduced wasps, in some treatments, but we also did not examine potential impacts of treatments on parasitism rates in nearby untreated trees. Thus, it is possible that parasitoid activity in the following summer after our treatments, and woodpecker activity in the following winter, was focused on untreated trees where larval densities were higher. *Phasgonophora sulcata* is univoltine and emerges later than EAB adults to target early instar larvae that have recently hatched (Roscoe et al. 2016). *Tetrastichus planipennisi* and *S. galinae* are multivoltine and emerge in late spring, before EAB emergence, to target late instar larvae (Jones et al. 2020). *Phasgonophora sulcata* and *T. planipennisi* are endoparasitoids and may be exposed to greater pesticide concentrations as the larvae they parasitize continue to feed, in comparison to *S. galinae* and *Atanycolus* spp. which are ectoparasitoids that paralyze larvae during oviposition (Bauer et al. 2015). Since *P. sulcata* targets early instar larvae and resides within larvae longer than *T. planipennisi*, the former species is likely exposed to greater pesticide concentrations. Additionally, early instar EAB larvae are typically more susceptible to insecticides compared to late instars (McCullough et al. 2011), increasing the likelihood of *P. sulcata* mortality within newly parasitized hosts, whereas third and fourth instars may be less likely to die after parasitism by *T. planipennisi*. Diamide treatments in late spring may kill early instar larvae within treated trees, allowing emerged *P. sulcata* to focus on early instars in untreated trees, while nonnative wasps could target surviving late instars in treated or nearby untreated trees. Conversely, treatments in late summer or early fall may kill early instar larvae that were recently parasitized by *P. sulcata*. Whether or not a reduction in *P. sulcata* densities within late summer treated trees is substantial enough to lower the species population within an infested stand, and thus limit parasitism of EAB larvae in nearby untreated trees, is unclear.

Drench applications of chlorantraniliprole may produce additive benefits alongside parasitoid releases to reduce EAB populations. For diamides to become more widely used for EAB management, it will be important to determine how long treatments can protect trees, where the product becomes concentrated within trees, what is the maximum tree size that can be protected, and if spring applications or other application methods yield greater efficacy.

## Supplementary Material

ieag103_Supplementary_Data

## Data Availability

Data used in this manuscript will be made available upon reasonable request to the corresponding author.
