# Supplementary material for: Influence of anthranilic diamide insecticides on emerald ash borer (Coleoptera: Buprestidae) larvae and establishment of introduced parasitoids in central Kentucky
Source: J Insect Sci. 2026 Sep 16;26(5):ieag103. doi: 10.1093/jisesa/ieag103 (PMC13581444; doi:10.1093/jisesa/ieag103)
Supplement: Supplementary file 1 [file ieag103_supplementary_data.docx]

**Influence of anthranilic diamide insecticides on emerald ash borer larvae and establishment of introduced parasitoids in central Kentucky.**

Fig. S1. Left: An ash tree with several recent woodpecker attack marks. Light colored wood where bark has been ripped away by woodpeckers indicates recent predation events. Right: An ash tree with many old woodpecker attack marks, indicated by the faded color of the wood where bark has been ripped away. All woodpecker marks that we counted as recent predation of EAB by woodpeckers looked similar to the attack marks in the left photo.

Table S1. Counts of living and dead EAB larvae across each development stage across all dissected logs (N = 164).

| Stage | Living | Dead | Total |
| --- | --- | --- | --- |
| L1 | 4 | 7 | 11 |
| L2 | 25 | 25 | 50 |
| L3 | 19 | 32 | 51 |
| L4 | 5 | 32 | 37 |
| J-larva or prepupa | 194 | 30 | 224 |
| Pupa | 20 | 6 | 26 |

Table S2. Average density of living and dead EAB (± S.E.) separated into early instars (L1 and L2) relative to later stages (L3, L4, J-larvae / prepupae, and pupae). Values are recorded as counts per surface area (m^2^) of debarked phloem. Asterisks next to values indicate significant differences (*P* < 0.05) relative to the control.

| Treatment | L1 and L2 living | L1 and L2 dead | L3 – Pupae living | L3 – pupae dead |
| --- | --- | --- | --- | --- |
| Chl_low | 3.2 ± 2.9 * | 2.2 ± 1.5 * | 10.8 ± 4.4 | 2.6 ± 1.3 |
| Chl_med | 0.7 ± 0.6 | 0.3 ± 0.2 | 7.7 ± 5.2 | 3.5 ± 1.8 |
| Chl_high | 0 | 0.2 ± 0.2 | 1.3 ± 0.5 * | 0.9 ± 0.4 |
| Cya_low | 1.0 ± 0.7 | 0.8 ± 0.6 | 5.7 ± 2.4 | 2.2 ± 1.5 |
| Cya_med | 0.4 ± 0.3 | 1.5 ± 0.8 | 4.3 ± 1.8 | 2.3 ± 1.3 |
| Cya_high | 0.7 ± 0.3 | 1.0 ± 0.5 | 5.1 ± 2.9 | 2.8 ± 1.6 |
| Control | 0.1 ± 0.1 | 0.2 ± 0.2 | 9.8 ± 4.1 | 3.8 ± 2.3 |
